# Epidemiological strategies for adapting clinical practice guidelines to the needs of multimorbid patients

**DOI:** 10.1186/1472-6963-13-352

**Published:** 2013-09-16

**Authors:** Eva Blozik, Hendrik van den Bussche, Felix Gurtner, Ingmar Schäfer, Martin Scherer

**Affiliations:** 1 Department of Primary Medical Care, University Medical Center Hamburg-Eppendorf, Martinistraße 52 D- 20246 Hamburg, Germany; 2 Swiss Federal Office of Public Health, Berne, Switzerland

**Keywords:** Clinical practice guideline, Multimorbidity, Chronic conditions

## Abstract

**Background:**

Clinical practice guidelines have been developed to improve the quality of health care. However, adherence to current monomorbidity-focused, mono-disciplinary guidelines may result in undesirable effects for persons with several comorbidities, in adverse interactions between drugs and diseases, conflicting management strategies, and polypharmacy. This is why new types of guidelines that address the problem of interacting medical interventions and conditions in multimorbid patients are needed.

**Discussion:**

Previous research projects investigated patterns of multimorbidity and were able to identify combinations of the most prevalent chronic conditions, or clusters of comorbidities. These results represent potential methodological starting points for the development of guidelines that account for multimorbidity. The objective of these efforts is to identify frequent reasons for interactions and adverse events that may occur when the current type of guideline is rigorously applied in multimorbid patients.

**Summary:**

The epidemiologic approaches described above may help guideline developers as a kind of check list of disease combinations that should systematically be considered during guideline development. Given the risk of worse outcomes in a huge group of vulnerable patients, researchers, guideline developers, and funding institutions should give first priority to the development of guidelines more appropriate for use in multimorbid persons.

## Background

The patients encountered in general practice, particularly those with multimorbidity, present a combination of interacting problems, which make adherence to clinical practice guidelines (CPG) and clinical decision making very difficult when recommendations cumulate across several conditions [1]. Nine out of ten patients seen in general practice have more than one chronic condition [2,3]. In 2005, Cynthia Boyd and colleagues illustrated the limitations of the current type of CPGs. They aggregated recommendations from relevant clinical guidelines for a hypothetical case of a 79-year-old multimorbid woman and ended up with 12 medications, prescribed in 19 partial doses, and a complicated nonpharmacological regimen [4]. This hypothetical case shows that the current way of designing CPGs is even associated with undesirable effects for persons with several comorbidities, and that adverse interactions between drugs and diseases, conflicting management strategies, and polypharmacy might result [5]. Polypharmacy exponentially increases the risk of experiencing adverse drug-drug or drug-disease interactions and is associated with adverse health outcomes such as mortality, hospitalisation, poor adherence, and geriatric syndromes (e.g. urinary incontinence, cognitive impairment, falls) [6-8].

Since the publication of Boyd’s study, it is widely acknowledged that elder persons and their health problems are not adequately addressed in current mainly mono-morbidity-focused and mono-disciplinary CPGs [5,9,10]. Various publications came to the conclusion that most CPGs do not provide guidance on the management of patients with comorbidities, especially for discordant combinations, [11] and it has been claimed repeatedly that CPGs should become explicit about the applicability of their recommendations to multimorbid patients [12-14]. Recently, Guthrie and colleagues suggested three strategies for adaptation of CPGs to take account of multimorbidity: 1) cross-referencing between CPGs using electronic delivery, 2) providing the suspected magnitude of benefits and harms of medical interventions recommended in CPGs, and 3) better using the existing evidence, e.g. by modeling the effects comorbidities may have on benefits and harms of treatments [15]. Although these adaptations are necessary, we do not consider them to be sufficient. For example, mere cross-referencing between CPGs would become hardly manageable in the presence of more than one comorbidity. The development of new CPGs that include multimorbidity-specific information is desirable. This article presents potential methodological starting points for identifying relevant comorbidities that should be taken into account during CPG development.

## Discussion

### Starting from epidemiologic data on prevalence of comorbidities

Given the fact that there are an almost indefinite number of possible disease combinations, [3] we suggest focusing on the most prevalent combinations of chronic conditions in multimorbid patients. The underlying assumption is that this would help to identify a large part of discrepant management strategies and interactions between treatments and conditions.

Epidemiologic data from previous studies may help to address either highly frequent combinations of chronic medical conditions or combinations particularly difficult to be handled [16]. For example, van den Bussche et al. found that triads of the six most prevalent individual chronic conditions (hypertension, hyperlipidemia, chronic low back pain, diabetes mellitus, osteoarthritis and chronic ischemic heart disease) cover nearly half on the elderly multimorbid population [3]. When developing or reviewing monomorbid CPGs, we propose to adapt the CPGs in the sense of adding recommendations on how to proceed in the management of the most prevalent associated conditions. For example, a CPG on the management of chronic ischemic heart disease should comment on treatments for hypertension, hyperlipidemia, chronic low back pain, diabetes mellitus, and/or osteoarthritis that interfere with the index disease. The most prevalent combinations of clinical conditions related to the index disease would thus be covered by the CPG.

Other analyses used explorative techniques such as cluster analysis [17] or factor analysis [16] to identify multimorbidity patterns. For example, Schäfer et al. investigated a list of 46 ICD10-based chronic conditions and identified overlapping clusters of 1) cardiovascular and metabolic diseases, 2) anxiety, depression, somatoform disorders and pain-related morbidity, and 3) neuropsychiatric disorders [17]. Based on such findings, CPGs might be supplemented with information on frequent interactions between treatments for diseases within one cluster. They could also address problems that may occur in patients with concurrent morbidities attributed to multiple clusters. For example, CPGs for cardiovascular diseases that recommend anticoagulants, antihypertensive, or lipid-lowering drugs (cluster 1) should comment on what should be done if a patient concurrently takes antidepressants, analgesics (to treat conditions included in cluster 2), or neuropsychiatric drugs (to treat conditions included in cluster 3) as interactions between these drugs are frequent or potentially serious.

### Applying clusters and triads using the example of Boyd’s case

Using the 10 most frequent triads of comorbidities as a basis for CPG development would mean that 5 of 9 conditions of the theoretical Boyd’s case would have been covered. In contrast, all of the comorbidities present in Boyd’s case are included in either the cardiovascular and metabolic disorder cluster or the anxiety, depression, somatoform disorders and pain cluster. Conditions from the neuropsychiatric disorders cluster are not present in Boyd’s case. Thus, the triad model may be used as a kind of checklist for frequent comorbidities, whereas the cluster model may be helpful for grouping recommendations related to comorbidities (Table 1).

**Table 1:** Comparison of the comorbidities mentioned in Boyd’s case and the triads (van den Bussche 2011) and cluster (Schäfer 2010) model

**Boyd 2005 [**[4]**]**	**van den Bussche 2011 [**[3]**]**	**Schäfer 2010 [**[17]**]**
Hypertension	Included in 9 triads	Included in CMD cluster
Chronic heart failure	Not included	Included in CMD cluster
Stable angina	Chronic ischemic heart disease included in 3 triads	Chronic ischemic heart disease included in CMD cluster
Atrial fibrillation	Not included	Cardiac arrhythmias included in CMD cluster
Hypercholesterolemia	Lipid metabolism disorder included in 6 triads	Lipid metabolism disorder included in CMD cluster
Diabetes mellitus	Included in 3 triads	Included in CMD cluster
Osteoarthritis	Included in 3 triads	Included in ADS/P cluster
Chronic obstructive pulmonary disease	Not included	Included in ADS/P cluster
Osteoporosis	Not included	Included in ADS/P cluster

CMD: cardiovascular and metabolic disorder.

ADS/P: anxiety, depression, somatoform disorder, pain.

NPS: neuropsychiatric disorder.

### Recommendations for CPG development

1. Increased applicability for multimorbid populations to be an objective of the CPG

The CPG development team should explicitly agree on the objective to create a guideline that ought to be applicable in an as large as possible proportion of the multimorbid population. We are convinced that this would increase the awareness of the CPG development team of potential problems associated with concurrent comorbidities. It may also foster the systematic inclusion of geriatric expertise in the CPG development process.

2. Stratification of trial results by age and health factors

Evidence for or against individual medical interventions should be interpreted separately for the relatively healthy patient population and the subgroup of multimorbid patients. Specifically, benefit in terms of survival of one intervention in comparison to a potential increase in quality of life or lower risk of adverse events of another intervention may be weighed differently in the multimorbid as compared to the relatively healthy population and this fact may thus lead to different recommendations for the multimorbid as opposed to the relatively healthy patient population. Thus, existing effectiveness data can be stratified by age group or presence of certain morbidities, and guideline developers could explicitly state if they were not able to identify trial data related to older or multimorbid populations. Relevant strata of comorbidities may be detected by using the triad or cluster model.

3. Managing excessive complexity by focusing on context specific CPGs

The fundamental challenge is to produce CPGs that are not excessively long and complex. One solution may be to develop context or problem specific guidelines that provide information appropriate to certain groups of patients (i.e. specific for the index condition and a certain range of comorbidities). Triads and clusters can be used as a basis for developing case vignettes, which, in turn, help CPG developers to address critical interactions between diseases and therapies.

4. Cross-referencing to existing instruments

Additionally, there are a number of existing initiatives that aim at reducing problems with conflicting medications, such as the Medication Appropriateness Index, [18] Beer’s criteria, [19] the Priscus list, [20] the START/ STOPP initiative, [21] or various computer-based drug interaction tools [22]. These instruments have not been systematically been considered in current CPGs, but CPG developers may refer to such instruments to reduce duplications and work load.

5. Involvement of all professional groups and patient perspective

A broad range of disciplines and professions is involved in the care for multimorbid patients. CPG development teams are increasingly multidisciplinary; however, the patient perspective and geriatric working principles such as focusing on functional abilities, independence and quality of life are not systematically included in the CPG development process. The triad and cluster model might help to identify the appropriate range of disciplines and professions involved in the management of the frequent comorbidities.

6. International collaboration

Given the fact that a large part of CPG work is done by professional associations with a low budget and by voluntary health experts, strategies for adapting CPGs to multimorbidity should not exponentially increase the work that needs to be done. Apart from focusing on the most prevalent conditions, the complexity and work load could be reduced if international collaboration would be intensified. Clearly, for specific health care settings or patient populations, it is reasonable to interpret the evidence and to consent recommendations on a local level. However, in principle, synthesising the evidence is not specific to individual health systems and may be exchanged across countries. The EUnetHTA Joint Action [23] and the Guidelines International Network (G-I-N) [24], for example, are evidence that this type of international cooperation and exchange is feasible.

### Limitations and future research

Clearly, the existing epidemiologic research data related to patterns of multimorbidity are not perfect. For example, lists of chronic conditions used in previous research were mainly not weighted for the impact of the listed condition on quality of life, activities of daily living, or prognosis [25]. Future research needs to go beyond studying the prevalence of certain combinations, and should adjust for the extent of comorbidities to compromise function, quality of life, and life expectancy. As many diseases have common risk factors and symptoms and might require similar treatment and prevention strategies, it might be helpful to cluster therapeutic strategies as opposed to conditions [26].

Limitations are also related to the existing evidence base and to the way evidence in older persons is generated. Current trials rarely include older, multimorbid persons and insufficiently control for confounding factors such as greater disease severity, place of residence, comorbid conditions, or functional limitations [5]. However, drug regulatory authorities increasingly consider the problem of multimorbidity. For example, in 2011 the European Medicines Agency published its geriatric medicines strategy which states that medicines should be studied appropriately in the older population [27]. Specifically, trials are needed that include elderly and comorbid populations, test complex interventions such as appropriate prescribing measures and evaluate relevant endpoints in these populations, e.g. functional outcomes, quality of life, disability, or pain [28]. For weighing risks and benefits of therapeutic interventions, future trials should consequently report absolute effect estimates for benefit and harm including the time period after which the effect is expected to occur. For priority setting with respect to comorbidities that need to be studied, triads and clusters may also be helpful.

## Summary

In the absence of specific recommendations for older, multimorbid patients treatment decisions are mainly based on expert opinion rather than on scientific evidence. The epidemiologic approaches described above may constitute a scientific basis for CPG developers to systematically consider disease combinations that are highly prevalent in the multimorbid population. The objective of these efforts is to identify frequent reasons for interactions and adverse events that may occur when the current type of CPG is rigorously applied in multimorbid patients, which has major implications for the weighting of benefits and harms of recommended interventions. Case vignettes selected based on epidemiologic data may present a practical link between recommendations in CPGs and the heterogenetic nature of multimorbidity in clinical practice. Given these suggestions will increase the complexity of CPG development, international collaboration and cross-reference to existing instruments should be intensified to balance the additional workload.

CPGs are not intended to replace the setting of diagnostic, therapeutic, and preventive priorities on the individual patient-doctor level. However, it is intolerable that providing health care in compliance with current CPGs might result in worse outcomes and increased cost for a huge group of vulnerable patients. Researchers, guideline developers, and funding institutions should give first priority to developing such a new type of CPGs. This will require the joint effort of all related societies and specialties.

## Abbreviations

CPG: Clinical practice guideline; EUnetHTA: European network for Health Technology Assessment; G-I-N: Guidelines International Network; ICD10: 10th revision of the International Statistical Classification of Diseases and Related Health Problems; START: Screening Tool to Alert doctors to Right Treatments; STOPP: Screening Tool of Older People’s potentially inappropriate Prescriptions

## Competing interests

The authors declare that they have no competing interests.

## Authors’ contributions

All authors participated in the conception of the paper. EB drafted the manuscript. HVDB, FG, IS, and MS commented on revisions to the manuscript. All authors read and approved the final manuscript.

## Pre-publication history

The pre-publication history for this paper can be accessed here:

http://www.biomedcentral.com/1472-6963/13/352/prepub
